# KCNQ2-Related Neonatal Epilepsy Treated With Vitamin B6: A Report of Two Cases and Literature Review

**DOI:** 10.3389/fneur.2022.826225

**Published:** 2022-03-25

**Authors:** Greta Amore, Ambra Butera, Giulia Spoto, Giulia Valentini, Maria Concetta Saia, Vincenzo Salpietro, Francesco Calì, Gabriella Di Rosa, Antonio Gennaro Nicotera

**Affiliations:** ^1^Department of Human Pathology of the Adult and Developmental Age “Gaetano Barresi”, Unit of Child Neurology and Psychiatry, University of Messina, Messina, Italy; ^2^Department of Neuromuscular Disorders, Institute of Neurology, University College London, London, United Kingdom; ^3^Pediatric Neurology and Muscular Diseases Unit, Scientific Institute for Research, Hospitalization and Healthcare (IRCCS) Istituto Giannina Gaslini, Genoa, Italy; ^4^Department of Neurosciences, Rehabilitation, Ophthalmology, Genetics, Maternal and Child Health, University of Genoa, Genoa, Italy; ^5^Oasi Research Institute-Scientific Institute for Research, Hospitalization and Healthcare (IRCCS), Troina, Italy

**Keywords:** KCNQ2, neonatal epilepsy, vitamin B6, pyridoxine, pyridoxal 5 phosphate, pyridoxine-dependent epilepsy (PDE), pyridoxine-responsive epilepsy

## Abstract

Potassium Voltage-Gated Channel Subfamily Q Member 2 (KCNQ2) gene has been initially associated with “Benign familial neonatal epilepsy” (BFNE). Amounting evidence arising by next-generation sequencing techniques have led to the definition of new phenotypes, such as neonatal epileptic encephalopathy (NEE), expanding the spectrum of KCNQ2-related epilepsies. Pyridoxine (PN) dependent epilepsies (PDE) are a heterogeneous group of autosomal recessive disorders associated with neonatal-onset seizures responsive to treatment with vitamin B6 (VitB6). Few cases of neonatal seizures due to KCNQ2 pathogenic variants have been reported as successfully responding to VitB6. We reported two cases of KCNQ2-related neonatal epilepsies involving a 5-year-old male with a paternally inherited heterozygous mutation (*c.1639C*>*T*; p.Arg547Trp), and a 10-year-old female with a *de novo* heterozygous mutation (*c.740C*>*T*; p.Ser247Leu). Both children benefited from VitB6 treatment. Although the mechanisms explaining the efficacy of VitB6 in such patients remain unclear, this treatment option in neonatal-onset seizures is easily taken into account in Neonatal Intensive Care Units (NICUs). Further studies should be conducted to better define clinical guidelines and treatment protocols.

## Introduction

Potassium Voltage-Gated Channel Subfamily Q Member 2 (KCNQ2) gene, first described by Singh et al. (1), is located on chromosome 20q13.3 and encodes for the voltage-gated potassium channel subunit Kv7.2. It has been traditionally related to “Benign familial neonatal epilepsy” (BFNE). This clinical entity often occurs within the first 2 weeks of life and is characterized by tonic/clonic early-onset seizures with apneic episodes and autonomic manifestations. Generally, it presents a self-limiting course for weeks/months with a regular neurodevelopmental outcome (2–4).

Amounting evidence arising from innovative genetic testing, and particularly next-generation sequencing (NGS) techniques, has revealed that different pathogenic variants of the same gene are responsible for several epileptic phenotypes; in particular, KCNQ2-related neonatal epileptic encephalopathy (NEE) contributed to expanding the spectrum of KCNQ2-related epilepsies (5). This is a severe phenotype, with an onset during the first days of life of intractable seizures (usually focal tonic), variably evolving into seizure freedom or a worsening/relapsing of the epileptic attacks over time, and an inevitable, though variable, adverse neurodevelopmental outcome (4–6). The electroencephalogram (EEG) may show a burst-suppression pattern or multifocal epileptiform abnormalities with background attenuation. This phenotype is mostly due to *de novo* missense variants of KCNQ2 causing a dominant-negative effect leading to a loss or a reduction of the M-current (a slow activating non-inactivating potassium current modulating the resting membrane potential) and less commonly to a gain of function effect, but not to a loss of function (haplo-insufficiency) (4, 7–9).

First described by Hunt et al. (10), pyridoxine (PN) dependent epilepsies (PDE) are a heterogeneous group of autosomal recessive disorders associated with neonatal-onset seizures not well-controlled with anti-epileptic drugs (AEDs) but responsive to large daily doses of vitamin B6 (VitB6) (11). PDE are caused by inborn errors of PN metabolism, usually due to pathogenic variants of ALDH7A1 gene (antiquitin). ALDH7A1 encodes for the alpha-aminoadipic semialdehyde (a-AASA) dehydrogenase, a key enzyme in the lysine metabolism, eventually implicated in the activation of VitB6 (12). Moreover, pyridoxamine5'-phosphate oxidase (PNPO) and pyridoxal 5′-phosphate binding protein (PLPBP) genes [both involved in the homeostasis of the active form of PN, namely pyridoxal 5′-phosphate (PLP)], have recently been related to variants of PDE more responsive to PLP than PN (13, 14).

The phenotypic spectrum associated with ALDHE mutation include: (1) Classic PDE-ALDH7A1 (with a dramatic early onset of prolonged or recurrent seizures of different types); (2) Atypical PDE-ALDH7A1 (including a late-onset, or seizures initially responding only to AEDs and eventually controllable only with PN/PLP several months later, or folic acid-responsive seizures, anyhow associated with variable degree of neurocognitive outcomes) (15). Clinical diagnosis is based on the demonstration of seizures ceasing after PN treatment, even after the elimination of all AEDs, and re-occurrence after its withdrawal (11, 16).

In addition to genetic testing, elevated levels of a-AASA in plasma and urine, and of pipecolic acid in plasma, urine and cerebrospinal fluid (CSF), as well as a high plasma-to-CSF PLP ratio, can be suggestive for VitB6 disorders (17, 18). However, a clear interpretation of these results is complicated; in fact, to date, no studies on CSF PLP levels in healthy newborns or infants are available, hence reference ranges derive from neurologically abnormal children undergoing CSF metabolite measurement for diagnostic workup (18).

Interestingly, PN and PLP have been proven to be effective in several early-onset seizures, not meeting the diagnosis of PDE, but instead falling within the category of “Pyridoxine-responsive epilepsy” (PRE) (19).

In this regard, they have been increasingly used in the clinical practice as add-on treatments in early-onset epilepsies, and it should be noted that few reports are today available specifically on KCNQ2-related neonatal epilepsies trialed with VitB6 (either in its inactive or active form) with variable outcomes (Table 1). Herein, we reported two new cases of KCNQ2-related neonatal epilepsy who benefited from VitB6 treatment.

**Table 1:** Patients described in the literature who carry a KCNQ2 mutation and have been trialed with vitamin B6.

**References**	**Patient**	**Phenotype**	**Mutation**	**Sex**	**Perinatal and early history**	**Seizure onset**	**Seizure features**	**AEDs administered (response)**	**Type of vitamin B6/Response**	**EEG**	**Neuroimaging**	**Seizure and clinical outcome**	**Additional information**
Our patients	1	Pyridoxine responsive epilepsy	c.1639C>Tp.Arg547Trp Paternally inherited heterozygous mutation	M	Normal	2 d	Clonic szs, perioral cyanosis, revolving eyes and buccal automatisms	MDL (acutely effective) PB, VPA (partially effective)	PN i.m.100 mg/d (later switched to oral maintenance therapy)/ Successful electro-clinical response	Initial EEG: Abnormal After PN initiation: Normal	MRI (4 d and 4 mo): Normal	Sz-free after PN start Today (6 ys): still sz-free; global DD; mild autism-like features	ALDH7A1 sequencing: negative. No further genetic testing was performed
	2	Pyridoxine responsive epilepsy	c.740C>Tp.Ser247Leu *De novo* heterozygous mutation in exon 5	F	Normal	2 d	Myoclonic szs with rolling eye movements	PB (acutely ineffective), Several AEDS at onset (ineffective), VGB, FA (in addition to PLP—effective), CBZ (maintenance therapy)	PN/ Unsuccessful PLP (start at 36 d, p.o. 500 mg/d in 4–6 doses)/Immediate sz control up to 18 mo	Initial EEG: Abnormal After PN initiation: Abnormal	MRI (2 d): Normal	Sz-freedom for 18 mo (after PLP start at 36 d) Today (10 ys): discrete sz-control	ALDH7A1 and PNPO sequencing: normal
Millichap et al. (20)	3	EOEE	c.1009G>A p.Ala337Thr	NR	NR	5 mo	NR	LEV, CBZ, CLB, CLZ, FLB, PB, VPA (NR) EZO (szs began to decrease by the second week on 13 mg/kg/d)	PN/ Unsuccessful	Initial EEG: Abnormal After EZO initiation: Normal	NR	Sz decrease after EZO initiation Abnormal development	Sz proved refractory to multiple AEDs and PN as well
Sands et al. (21)	4–5 (twins)	BFNE	c.1057C>G p.Arg353Gl	F	Born at 34 wks	2 d	Focal asymmetric tonic posturing, associated with apnea and desaturation	PB i.v. 40 mg/kg, CLN p.o., DZP i.v. (NR) CBZ p.o. 10 mg/kg/day (effective)	PN i.v.100 mg/ Unsuccessful	EEG (patient 4): Normal EEG (patient 5): Abnormal	MRI: Normal	Both sz-free off meds at 16 ys Normal development	Family history positive for neonatal szs
Mulkey et al. (22)	6	NEE	c.601C>T p.R201C	NR	Normal	1 d	Exaggerated and sustained startle reaction to touch	EZO, VGB, CLZ, FA (NR)	PN, PLP/ NR	Initial and 1 mo EEG: Abnormal	MRI (1 wk and 3 mo): Abnormal	Sz outcome NR Profound DD Died at 13 mo for cardio-pulmonary arrest	Early neurologic exam: Abnormal
	7	NEE	c.601C>T p.R201C	NR	Normal	1 d	Exaggerated and sustained startle reaction to noise/touch, apnea Infantile spasms at 4 mo	EZO, VGB, PB, VPA, PHT, FA, TPM, LEV, ZNS, LOC, CBZ, STM, KD, CBD enriched cannabis (NR)	PN, PLP/ NR	Initial and 1 mo EEG: Abnormal	MRI (1 wk): Abnormal	Sz outcome NR Profound DD	Early neurologic exam: Abnormal
	8	NEE	c.601C>T p.R201C	NR	Normal	2 d	Stiffening events	VGB, PB, TPM, ACTH, KD, LEV, CLZ, FA (NR)	PN, PLP/NR	Initial and 1 mo EEG: Abnormal	MRI (1 wk): Normal MRI (1 y): Signal anomalies	Sz outcome NR Profound DD	Early neurologic exam: Abnormal
	9	NEE	c.601C>T p.R201C	NR	Born at term: bradycardia prior to delivery via caesarian section	1 d	Exaggerated startle response, apnea	EZO, VGB, CBZ, CLZ, PB, FA, PHT (NR)	PLP/NR	Initial and 1 mo EEG: Abnormal	MRI (2 and 4 mo): Abnormal	Sz outcome NR Profound DD Deceased	Early neurologic exam: Abnormal
	10	NEE	c.602G>A p.R201H	NR	Normal	1 d	Myoclonic spontaneous movements, exaggerated startle to noise/ touch Infantile Spasms at 2 months	VGB, CBZ, ZNS, PB, FA (NR)	PLP/NR	3 mo: Abnormal	MRI (3 mo): Abnormal	Sz outcome NR Profound DD	Early neurologic exam: Abnormal
Sharawat et al. (23)	11	NEE	c.835G >A p.Gly279Ser Likely pathogenic heterozygous Missense variant in exon 6	M	Normal	7 d	Repeated szs with up-rolling of eyeballs, generalized stiffening of body and cry	PB (sz-free for 3 months in combination with PN) CBZ 20 mg/kg/d (sz-free within a week)	PN started at 15 mg/kg/d, later increased to 50 mg/kg/d/Partial response	Initial EEG: Abnormal Repeat EEG: Normal	MRI (28 d): Normal	Sz-free at 3 mo (after PN start) 5 mo: re-occurrence of 1–2 sz/mo 1 y: re-occurrence of 8–10 sz/d Sz-free at last follow-up and mild global DD	Partial and temporary response to PN. Sz-freedom achieved with CBZ and PN together
Spagnoli et al. (24)	12	NEE	c.913_915del p.Phe305del *De novo*	M	Born full-term Apgar Score 3/10 Urgent cesarean section (cardio-tocographic abnormalities)	10 hs	10 hs: versive tonic spasms, ± flushing and desaturation ± focal clonic components	PB, PHT, LEV, MDL (ineffective) CBZ (sz-free)	PN, PLP/ Unsuccessful	Initial EEG: Abnormal After plural AEDS (9 mo): Abnormal	MRI (1 d): Minor abnormalities	Sz-free at 9 mo (after CBZ start) Severe DD; Spastic-dystonic tetraplegia	The patient was trialed with FA as well, unsuccessfully
Vilan et al. (25)	13	BFNE	c.1076C>A p.Thr359Lys Maternally inherited	F	Normal	1 d	Tonic with cyanosis	PB, CZP, PHT, VPA (NR) Lidocaine (partially effective)	PN/ Unsuccessful	Ictal aEEG: Abnormal Interictal: Normal	MRI: Normal	Sz-free at 1.5 mo Recurrence of frequent Sz after 4 years; Mild ID, ADHD (13 y)	Pyridoxine was administered to 8 infants without any beneficial effect. All infants needed ≥2 AEDs to control their szs
	14	NR	c.1955dupC p.Pro652fs *De novo*	M	Normal	2 d	Tonic with cyanosis	PB, MDL, CZP (NR) Lidocaine (partially effective)	PN/Unsuccessful	Ictal aEEG: Abnormal Interictal: Normal	MRI: Normal	Sz-free at 21 d Recurrence of 2 Sz at 4 ys. Mild delay in MD (5 ys)	
	15	NR	c.1065C>G p.Asp355Glu *De novo*	M	Normal	2 d	Tonic with cyanosis	PB, MDL (NR) Lidocaine (acutely effective) PHT (effective)	PN/Unsuccessful	Ictal aEEG: Abnormal Interictal: Normal	MRI: Normal	Sz-free at 12 d Normal outcome (2 ys)	
	16	NR	c.2296delC p.Leu766fs *De novo*	M	Normal	3 d	Tonic with cyanosis	PB, MDL (NR) Lidocaine (partially effective)	PN/Unsuccessful	Ictal aEEG: Normal Interictal: Discontinuous normal voltage	MRI: Normal	Sz-free at 14 d Further outcome: NR	
	17	NR	c.1527delA p.Glu509fs *De novo*	M	Normal	24 d	Tonic with cyanosis	PB (not effective)	PN/Unsuccessful	Ictal aEEG: Abnormal Interictal: Normal	MRI: Normal	Sz-free at 14 d Further outcome: NR	
	18	NEE	c.830C>T p.Thr277lle *De novo*	M	Normal	2 d	Tonic with cyanosis followed by focal clonic activity	PB, MDL, CZP, TPM (NR) LEV and lidocaine (acutely effective) VPA (effective)	PN/Unsuccessful	Ictal aEEG: Abnormal Interictal: Discontinuous normal voltage	MRI: Normal	Sz-free at 1.5 mo Non-verbal, autistic features, delay in MD (3 ys)	
	19	NR	c.1657C>T p.Arg553Trp *De novo*	F	Normal	1 d	Tonic with cyanosis followed by focal clonic activity	PB, LEV, MDL (NR) CBZ (effective)	PN/Unsuccessful	Ictal aEEG: Abnormal Interictal: Normal	MRI: Normal	Sz-free at 15 d Normal outcome (16 mo)	
	20	NEE	c.901G>A p.Gly301Ser *De novo*	F	Normal	1 d	Tonic with cyanosis	PB, MDL, LEV (NR) Lidocaine (acutely effective) CBZ (effective)	PN/Unsuccessful	Ictal aEEG: Abnormal Interictal: Normal	MRI: Signal anomalies	Sz-free at 15 d	
Pisano et al. (26)	21–32	NEE	12 patients were trialed with adequate dose of pyridoxine, 7 received pyridoxal-phosphate. At the onset, all patients showed axial hypotonia. During follow-up, cognitive impairment										
Klotz et al. (27)	33	NEE	c.1023G>C p.Gln341His *De novo*	F	Born at term but SGA	7–8 d	Tonic with cyanosis.	PB, LEV (partially effective, in combination with PN)	PN trial: 30 mg/kg over 3 d/ No immediate effect on sz frequency or EEG Consequent PLP trial: 30 mg/kg/Improvement of sz frequency shortly after its start (in combination with PB and LEV)	Initial EEG: Abnormal Last EEG (date NR): Abnormal	MRI: Normal	Sz reduction (once-twice every few wks) Neuro-cognitive sequelae: abnormal MD and DD	No detected mutations in ALDH7A1 or PNPO After PLP discontinuation: re-occurrence of 20 szs/d After PLP reintroduction seizures resolved Further switch to PN: no change in seizure frequency
Reid et al. (18)	34	NEE?	c.629G>A p.Arg210His *De novo*	F	Born at 38 wks + 6 by spontaneous labor, after an uneventful pregnancy	4 d	Episodes of choking and cyanosis, associated with stiffening after which she became floppy; “cycling” movements of arms; desaturation	PB, LZP (not effective) PHT, CBZ (partially effective in combination with PN)	PN, PLP/Successful 2 mo: sz control after 6 d of 0.25 mg of PN (oral drops), at a time when she was also receiving 6.7 mg/kg/day of PHT and 11 mg/kg/day of CBZ	9 d: Abnormal 7 ys: Abnormal	MRI (28 d); Minor abnormalities	Sz outcome at 7 years: szs presenting only during intercurrent illness 7 y: DD with minimal expressive language	No mutations detected in ALDH7A1 or PNPO
Allen et al. (3)	35	BFNE	c.419_430dupp.Val143_Arg144ins GlnTyrPheVal Maternally inherited (affected mother)	F	Normal	4 d	Clonic and tonic szs. Clusters multiple/day or days sz-free. Minor cyanosis subsequently, mainly tonic. Multiple/day, then weeks and months sz–free	LEV (some response, required dose increases) Other drugs used but ineffective: PB, MDZ, LZP	PLP (used acutely)/ NR	Initial EEG: Abnormal 3.5 mo: Normal	MRI (3 wks): Normal	Sz outcome: sporadic breakthrough minor szs Normal developmental outcome (1 y)	
Mefford et al. (16)	36	Pyridoxine-dependent epilepsy?	1.5 Mb terminal deletion of the long arm of chromosome 20	M	Precipitous after a 36 week pregnancy complicated by frequent Braxton-Hicks contractions.	2 wks	Reddening and tonic stiffening of arms, lasting approximately 1 min Initially sporadic but, by 8 wks of age, occurring 4–6/day	PB 15 mg/day (partial response)	PN 100 mg/d, later increased to 200 mg/d and eventually reduced to 150 mg/d/ Good electroclinical response (so that PB was discontinued at 11 months)	Initial EEG: Abnormal 5 ys: Abnormal	First MRI: Minor abnormalities MRI (11 mo): Normal	Sz-free (7 ys) ID and delay in MD	Sequencing of the ALDH7A1 gene did not detect mutations
Weckhuysen et al. (5)	37	NEE	c.613A>G p.Ile205Val	M	During the last 2 months of pregnancy rhythmical jerking similar to szs Subsequent normal perinatal and early development	2 d	Generalized tonic with clonic components, lip smacking, back arching, and apnoea. Multiple szs daily	VGB (initially reduced szs and normalized EEG with 7 wks sz freedom) MDZ (partially effective) PB, FA, betamethasone, VPA (all ineffective) TPM, VGB (effective in combination with PN)	PN/The combination of TPM, VGB, and PN controlled szs	7 d: Abnormal 9 mo: Normal	CT scan (2 d) and MRI (11 d and 3.5 ys): Signal anomalies	Status epilepticus at 3 mo; Sz -free from 9 mo until 8 ys ID and delay in MD	
Borgatti et al. (28)	38	BFNE, epileptic encephalopathy, and profound mental retardation?	c.1620G>A p.K526N Maternally inherited (Affected mother and two younger sisters)	F	Born at 40 wks by cesarean section due to podalic presentation	3 d	Clonic szs. Subsequently right sided clonic and tonic-clonic szs with oro-alimentary automatism	ACTH (partially effective) PB, VGB, benzodiazepines, PHT, VPA, CZP, immune-globulin (ineffective)	PN/NR	Initial ictal EEG: Abnormal Last EEG (date NR): Abnormal	MRI (around 4 mo): Abnormal	Not achieved complete sz control. Many polymorphic szs/d Severe spastic tetraparesis and profound ID	
Dedek et al. (29)	39	BNFE	p.Ser247Trp	M	Born by cesarean section due to prolonged delivery period and symptoms of fetal distress	3 d	Left or right head deviation, and upper and lower limb involvement	ACTH (effective) PB, PHT,VGB (ineffective)	PN /Unsuccessful	8 d: Normal 2.5 ys: Abnormal	CT (7 d): Normal MRI (41 d): Normal	Sz-free (szs stopped at 13 wks) Immediate improvement of EEG after ACTH initiation DD (2 ys and 5 mo)	
Martin et al. (30)	40	NEE (Ohtahara syndrome)	c.827C>T p.T276I	M	Born at 41 wks by emergency cesarean section due to failure to progress	1 d	Cyanotic episodes, then more obvious szs; tonic spasms	TPM, DZP, and NZP (fits initially continued, but at 5 months were less severe, ceasing by 17 months) CZP, VGB, FA (ineffective)	PLP/ Unsuccessful	1 d: Abnormal 4 ys: Abnormal	MRI: Abnormal	Sz-free Severe DD (4 ys)	
Numis et al. (31)	41	NEE	c.1734 G>Cp.Met578Ile	NR	Born at 34 wks. Lack of visual fixation, decreased spontaneous movements, and axial hypotonia	4 d	Tonic head, conjugate eye, mouth deviation, unilateral tonic abduction of the limbs. Apnoea and desaturation	CBZ (effective—sz free within 2 wks) PB, LEV, TPM, VGB, CLB, CZP, KD, FA (ineffective)	PN, PLP/ Unsuccessful	Interictal and Ictal EEG: Abnormal	MRI (20 and 33 d): Abnormal	Sz-free DD and delay in MD	
Saitsu et al. (32)	42	NEE (Ohtahara syndrome)	c.1010C>G p.A337G	M	NR	7 d	Tonic szs, vomiting. Complex partial szs since age 5	High dose PB (sz-free and burst-suppression disappeared) ZNS (ineffective)	PN/ Unsuccessful	Initial EEG: Abnormal	NR	Sz-free after high dose PB DD and delay in MD	
	43	NEE (Ohtahara syndrome)	c.341C>T p.T114I	F	NR	0 d	Tremor of the upper extremities then generalized convulsions with cyanosis Complex partial szs since age 5	ZNS (sz free) CZP, PHT (ineffective)	PN/ Unsuccessful	Initial EEG: Abnormal	NR	Sz-free after ZNS Profound DD and abnormal MD	
	44	NEE (Ohtahara syndrome)	c.794C>T p.A265V	M	NR	1 d	Apnoeic spell, then tonic spasms with right opsoclonus like movement.	ZNS, VPA, CZP, CBZ (ineffective)	PN/ Unsuccessful	Initial EEG: Abnormal	NR	Intractable szs DD Myoclonus at the bilateral upper extremities	
Kato et al. (7)	45	NEE (Ohtahara syndrome)	c.650C>Ap.Thr217Asn *De novo*	F	NR	0 d	At onset: Pale face for tens of seconds 1 d: Eye deviation to left followed by tonic szs (0.5–1/h)	High dose PB (sz-free) ZNS (ineffective)	PLP/ Unsuccessful	1 d: Abnormal	MRI (2 d, 1 and 6 mo): Signal anomalies MRI (2 ys): Normal	Sz-free after high dose of PB Profound DD and abnormal MD	
	46	NEE?	c.794C>T p.Ala265Val *De novo*	M	NR	2 d	At onset: Facial flushing and eye fixation 3 d: Tonic szs (daily)	DZP, MDL, high-dose PB (partially effective) VPA (ineffective) CBZ (successful)	PLP/ Unsuccessful	5 d:Abnormal	MRI (7 mo and 2 y): Abnormal	Sz-free after CBZ. No szs since 16 mo Moderate DD and abnormal MD	
	47	NEE (Ohtahara syndrome)	c.794C>T p.Ala265Val *De novo*	M	NR	2 d	At onset: No cry, poor suck, stiffening, and arching with eye rolling 5 d: left-sided szs	PB, CLB, MDZ, VGB (ineffective)	PLP/ Unsuccessful	5 d:Abnormal	MRI (0 mo): Normal	Intractable szs DD and abnormal MD Died at 3 mo	
	48	NEE (Ohtahara syndrome)	c.854C>A p.Pro285His Maternally inherited	F	NR	0 d	At onset: poor feeding followed irritability with hypoxia and tonic szs (1–4/d).	VPA (sz-free) PB (ineffective)	PLP/ Unsuccessful	12 d:Abnormal	MRI (12 d and 3 mo): Signal anomalies	Sz-free after VPA (since 3 mo) DQ 35. Moderate DD and abnormal MD	
	49	NEE (Ohtahara syndrome)	c.881C>T p.Ala294Val *De novo* Domain in protein: Transmembrane domain (S4)	M	NR	1 w	Convulsion-like movements followed by asymmetric tonic szs	TPM (sz-free) ZNS, VPA (partially effective) CZP, PB (ineffective)	PLP/ Unsuccessful	<1 mo: Abnormal 3 mo: Abnormal	MRI (3 mo and 9 mo): Abnormal	Sz-free since 6 mo Profound DD and abnormal MD	
	50	NEE (Ohtahara syndrome)	c.997C>Tp.Arg333Trp *De novo* C-terminal region	M	NR	2 d	Tonic szs followed by partial ones (eyes rolling up)	ZNS (almost sz-free) VPA, lidocaine (partially effective) DZP, PB, PHT (partially effective)	PLP/ Unsuccessful	42 d:Abnormal	NR	Sz-free. Only one sz in 10 ys. Severe DD	
	51	NEE (Ohtahara syndrome)	c.1689C>Gp.Asp563Glu C-terminal region	F	NR	1 d	Poor feeding with cyanosis, followed by tonic szs, facial clonic sz, and generalized tonic-clonic convulsions	CBZ and CZP (sz-free) PHT, PB (partially effective) VPA, NZP (ineffective)	PLP/ Unsuccessful	4 d: Abnormal	CT (0 m): Normal	No szs since 10 y, but relapsed at 24 ys after a y of drug withdrawal Moderate DD with autistic features	
Milh et al. (6)	52–57	Six patients described that have been treated with vitamin B_6_ during the first month of life. Responses to each AED not stated											

*NR, not reported; BFNE, Benign familial neonatal epilepsy; EIEE, early infantile epileptic encephalopathy; EOEE, early onset epileptic encephalopathy; NEE, neonatal epileptic encephalopathy; F, female; M, male; IV, intravenous; P.O., per os; S, second(s); Mn, minute(s); H(s), hour(s); D, day/s; Wk(s), week(s); Mo, month(s); Y(s), year(s); CBZ, carbamazepine; CLB, clobazam; CF, calcium folinate; CLZ, clonazepam; EZO, ezogabine; FA, folinic acid; FLB, felbamate; KD, ketogenic diet; LEV, levetiracetam; LOC, lacosamide; LZP, lorazepam; MDL, midazolam; NZP, nitrazepam; PB, phenobarbital; PHT, phenytoin; PN, pyridoxine; PLP, pyridoxal 5′ phosphate; TPM, topiramate; STM, sulthiame; VB6, vitamin B6; VGB, Vigabatrin; VPA, valproic acid; ZNS, zonisamide; Sz(s), seizure(s); DD, developmental delay; ID, intellectual disability; MD, motor development*.

## Case Reports

### Case 1

A 6-year-old Caucasian boy was born at term by eutocic vaginal delivery from an uneventful pregnancy. Birth weight was 2,870 gr. Perinatal period was unremarkable and physical examination at birth normal. He was the second child of healthy related parents with common ancestors. The patient's family history was positive for epilepsy both in the maternal and paternal line; in particular, the father was diagnosed with a KCNQ2-related-epilepsy treated with Valproate (VPA) and Phenobarbital (Pb). It is to note that the father referred this data only at the end of our patient's genetic investigation.

On the second day of life, the patient presented clonic seizures at the limbs. The episodes resolved spontaneously. On the fourth day, he was hospitalized for the re-occurrence of seizures associated with perioral cyanosis. An electroencephalogram (EEG) showed polyspike wave complexes. Given the persistence of continuous clonic seizures, associated with perioral cyanosis, revolving eyes and buccal automatisms (sucking), midazolam by continuous intravenous infusion and intravenous boluses of Pb were administered. Brain magnetic resonance imaging (MRI) was normal. On the 10th day of life, oral Pb treatment was started, with seizure control for almost 3 months. Meanwhile, metabolic tests and the sequencing of KCNQ2 gene were performed.

At 3 months of life, recurrent convulsive seizures/status epilepticus occurred; thus, VPA was started at 10 mg/kg/bid, with subsequent titration up to 30 mg/kg/bid with transient remission of seizures. About a month later, focal clonic seizures at the limbs appeared, mainly with deviation of the eyes and cyanosis, lasting 60 s. An EEG showed “slight abnormalities of electrical brain activity in the left posterior areas.” Further brain MRI scans were normal. Given the age of the patient and the partial response to stabilized treatment with VPA (100 mg/bid) and intravenous Pb (7.5 mg/bid), we decided to start PN supplementation as an intramuscular formulation of the vitamin B complex, containing 100 mg/day of PN. Seizure cessation and disappearance of the EEG abnormalities were gained after few days of treatment. Maintenance treatment was carried on, in agreement with the patient's parents (who were hesitant about PN withdrawal, as initially planned), with an oral formulation of multivitamin B complex at a very low dosage of 5 ml/day, equivalent to 0.45 mg of PN, with a seizure-free interval of about a month. In the meanwhile, a Developmental Quotient (DQ) of 70 was measured by using the Brunet-Lezine developmental scale, detecting a neurodevelopmental delay. The next month, in concomitance to an infectious episode, he presented with a further epileptic seizure characterized by clonic movements of the limbs, revolving eyes, perioral cyanosis, lasting about 60 s and resolving spontaneously. Antiepileptic treatment was unchanged. No further seizures occurred thereafter, either during infectious episodes.

Throughout the following years, VPA first, and Pb later, were discontinued. Today the child is seizure-free and takes daily multivitamin B complex, also containing PN. Despite the global neurodevelopmental delay, he is able to walk alone, jump and climb; his expressive language is limited to simple and short phrases and characterized by dyslalia, whereas the comprehension is adequate. The child presents mild autism-like features (such as self-stimulatory, repetitive and stereotyped behaviors, and relational difficulties). He has also gained sphincteric control.

**Figure 1:**
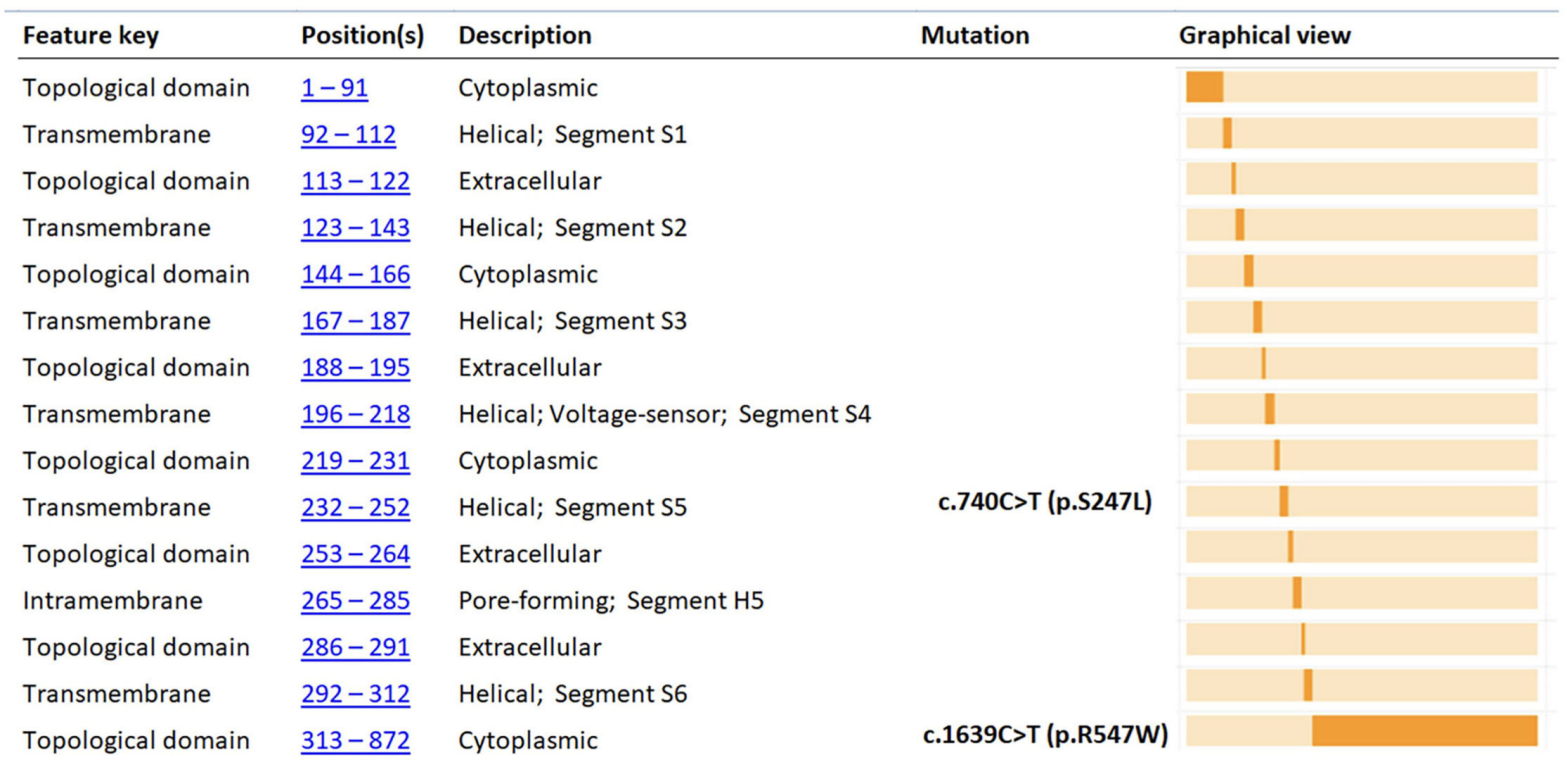
Functional domain position of p.R547W and p.S247L amino acid changes of KCNQ2 protein (adapted from https://www.uniprot.org). The figure shows the KCNQ2 functional domain position of the variants found in our two cases (p.R547W and p.S247L). *In silico* analysis (Table 2) predicted that the two mutations have potential strong damaging effect on the structure/function of the KCNQ2 protein.

Direct sequencing of the KCNQ2 (NM_172107.4; NP_742105.1) gene demonstrated a paternally inherited heterozygous mutation (c.1639C>T; p.Arg547Trp), leading to the replacement of Arginine with Tryptophan at position 547 of the protein and involving a conserved amino-acid of the protein predicted to have functional consequences on the protein (Figure 1). This variant had already been reported in the literature by Zara et al. (2) (as causative of a BFNE in a female patient with a maternal inheritance) and Lindy et al. (33) (no clinical data available). *In silico* analysis performed with bioinformatic tools predicted that mutation has a potential strong damaging effect on the structure/function of the KCNQ2 protein. ACMG standard criteria for assessment of pathogenicity of variants (Varsome) (34) should be considered as “Pathogenic” (Table 2).

**Table 2:** *In silico* prediction of the KCNQ2 missense mutations^*^.

**NM_172107.4 (NP_742105.1) (GRCh38)**	**c.1639C>T (p.R547W)**	**c.740C>T (p.S247L)**
PolyPhen2 prediction: B (benign), P (possibly damaging), D (probably damaging)	D	Variable: P;P;B;B;B;B
SIFT prediction	Damaging	Damaging
LRT prediction	Deleterious	Neutral
MutationTaster prediction	Disease causing	Disease causing
MutationAssessor prediction	Medium impact	High impact
FATHMM	Damaging	Damaging
fathmm-MKL	Damaging	Damaging
M-CAP	Damaging	Damaging
CADD [the larger the score the more likely the SNP is damaging (PHRED-like)]	27.2	25.9
MetaSVM	Damaging	Damaging
MetaLR	Damaging	Damaging
PhyloP 20way the larger the score, the more conserved the site (max 1.199000)	−0.409000	0.982000
PhyloP 100way the larger the score, the more conserved the site (max 10.003000)	0.097000	6,62,8000
GERP RS the larger the score, the more conserved the site (max 6.17).	−1.15	3.25
1000 Genomes	No data	No data
gnomAD	No data	No data
Interpro domain	Potassium channel, voltage dependent, KCNQ, C-terminal	Ion transport domain
ClinVar interpretation	Pathogenic/Likely_pathogenic	Pathogenic/Likely_pathogenic
ACMG classification (Varsome**)	Pathogenic	Pathogenic

*Data from HGMD professional database [www.hgmd.cf.ac.uk; (35)].

**Kopanos et al. (34).

In the case of our patient, ALDH7A1 sequencing was later reported to be negative, and no further genetic testing was performed. This data prevented us the possibility to explain his positive response to PN and his clinical phenotype (that, despite the clinical continuum existing between BFNE and NEE, seems closer to the NEE phenotype), in which a possible role of other genes on his impaired neurodevelopment cannot be ruled out.

### Case 2

A 10-year-old Caucasian female was born at term by eutocic vaginal delivery from an uneventful pregnancy. Birth weight was 3,400 gr. Prenatal and perinatal history was unremarkable. On the second day of life, the baby showed myoclonic seizures associated with sudden loss of muscle tone and rolling eye movements. The patient started treatment with Pb, without effects. The EEG showed a suppression-burst pattern. Brain MRI and routine metabolic investigations resulted to be normal. At day 36 of life, after unsuccessful therapeutic attempts with Pb and VPA, and the start of PN administration, she was admitted to the NICU without considerable response. Herein, she was started on oral PLP therapy (up to a dose of 500 mg/day divided in 4–6 administrations), achieving immediate seizure control. An extended metabolic workup was performed highlighting an elevation of pipecolic acid both in serum and urine, albeit the sequencing of ALDH7A1 and PNPO genes was normal. Given the good clinical response to PLP and the poor neurodevelopment performance (no achievement of motor milestones, marked muscular hypotonia, and visual disturbances), she received a diagnosis of “Focal epilepsy with pyridoxal phosphate-responsive seizures and global neurodevelopmental delay.”

After 18 months of seizure-free interval, epileptic tonic spasms occurred, both during sleep and wakefulness. The EEG showed multifocal epileptic discharges.

Low CSF folic acid levels were revealed; hence, folic acid was started (7.5 mg/bid, later increased at 7.5 mg/tid), in add-on to her previous therapy, with benefits in terms of number and intensity of epileptic seizures.

Despite the overall discrete seizure control, subsequent interictal EEG recordings showed marked diffuse abnormalities up to a *quasi*-periodic epileptiform pattern. The patient showed severe global neurodevelopmental delay at the follow-up.

Further genetic testing was performed. Array-CGH was not informative. Whereas, trios NGS (epilepsy panel) revealed a *de novo* heterozygous mutation in Exon 5 of the KCNQ2 gene (NM_172107.4; NP_742105.1): c.740C>T/(p.Ser247Leu), affecting a highly conserved aminoacidic region and predicted to be deleterious *in silico* programs (Figure 1). However, using the standard procedures for assessment of pathogenicity of variants (ACMG criteria—Varsome) (34), should be considered as “Pathogenic” (Table 2). This variant has already been reported in the literature and related to early-onset epileptic phenotypes with a variable degree of neurocognitive impairment (36–41). However, none of these reports shows evidence of VitB6 supplementation. Our patient was maintained on Vigabatrin, PLP, and Folic acid for several years. By the age of 10 years, given the occurrence of severe behavioral and sleep disorders, PN, Folic acid, and Vigabatrin were withdrawn, and maintenance therapy with carbamazepine, in add-on to Lorazepam and Promazine, was started.

To date, the child failed to achieve trunk control, independent standing or walking. She shows axial hypotonia and limbs hypertonia. Language is limited to vocalizations and she presents severe intellectual disability. In addition, behavioral disturbances are evident, with aggressive manifestations and sleep disorders. She displays a good clinical control, with occurrence of rare seizures (one episode/1–2 years).

## Discussion

Herein, we reported two cases of KCNQ2-related epilepsy, a 5-year-old male with a paternally inherited heterozygous mutation (c.1639C>T; p.Arg547Trp), and a 10-year-old female with a *de novo* heterozygous mutation (c.740C>T; p.Ser247Leu), who benefited from PN treatment.

The first patient presented with neonatal epilepsy with an onset during the first week of life, which was later ascribed to a KCNQ2 pathogenic variant paternally inherited.

The same variant had already been described in the literature (2, 33) and reported as causative of a BFNE phenotype in a female patient with maternal inheritance (2).

Our patient, with his overall good seizure-responsivity despite impaired neurodevelopment, does not show all the typical characteristics of BFNE or NEE. Even if no evidence of an inborn error of VitB6 was available for our patient (ruling out the diagnosis of a PDE), his seizures, scarcely controlled by AEDs, revealed a good and immediate electro-clinical response to the initiation of intramuscular PN. Interestingly, this effect did not cease after switching to PN oral administration, neither after the discontinuation of AEDs, strengthening the diagnostic hypothesis of PN-responsive epilepsy (PRE).

The second patient, after a sudden and severe onset of neonatal seizures, requiring plural hospitalizations, and failed attempts with AEDs, such as Pb and VPA, and with PN too, demonstrated an immediate clinical benefit from the initiation of PLP, and, over time, to folic acid as well.

In this case, a *de novo* disease-causing KCNQ2 variant was detected (c.740C>T; p.Ser247Leu), and the good clinical response to PLP and folic acid were supported, respectively, by elevated levels of pipecolic acid both in serum and urine and by reduced CSF folates level. Moreover, genetic testing resulted negative for ALDH7A1 and PNPO pathological variants. These data supported the possibility to classify this clinical phenotype into the PRE spectrum.

Moreover, even though the clear effect of PLP reduced over time, other AEDs (i.e., Pb and VPA) proved to be ineffective, and the neurocognitive outcome of the patient is quite poor, she still presents an overall good clinical control, with seizures being years apart.

Summarizing, regardless of the several divergences between these 2 cases, both of them may fit into the category of PRE, particularly within the subgroup of KCNQ2-related ones.

Data from the literature have demonstrated the usefulness of VitB6, either as PN or, even better, as PLP, in certain types of neonatal-onset epilepsies (13, 19). In addition, recent reports have been unraveling a potential correlation between KCNQ2-related epilepsies and VitB6 responsiveness (18). To date, several cases of patients carrying a pathogenic variant of KCNQ2 trialed with VitB6 (either in the form of PN or PLP) have been reported in the literature. Unfortunately, the effects of VitB6 have not been accurately described for all of them. Additionally, several cases have been extracted from studies focusing on other than the KCNQ2-PN correlation, making it arduous to infer the exact impact of VitB6 on the clinical picture (6, 22, 26). Five reports have openly disclosed various degrees of a successful response to PN/PLP in KCNQ2-related epilepsies (5, 16, 18, 23, 27). Interestingly, despite their small number, all patients share a neuro-cognitive impairment in the lack of genetic data confirming/denying the PN-dependency/responsiveness, in line with ours. All cases are reported in Table 1 (and detailed in the complete version of the table—see Supplementary Material).

**Figure 2:**
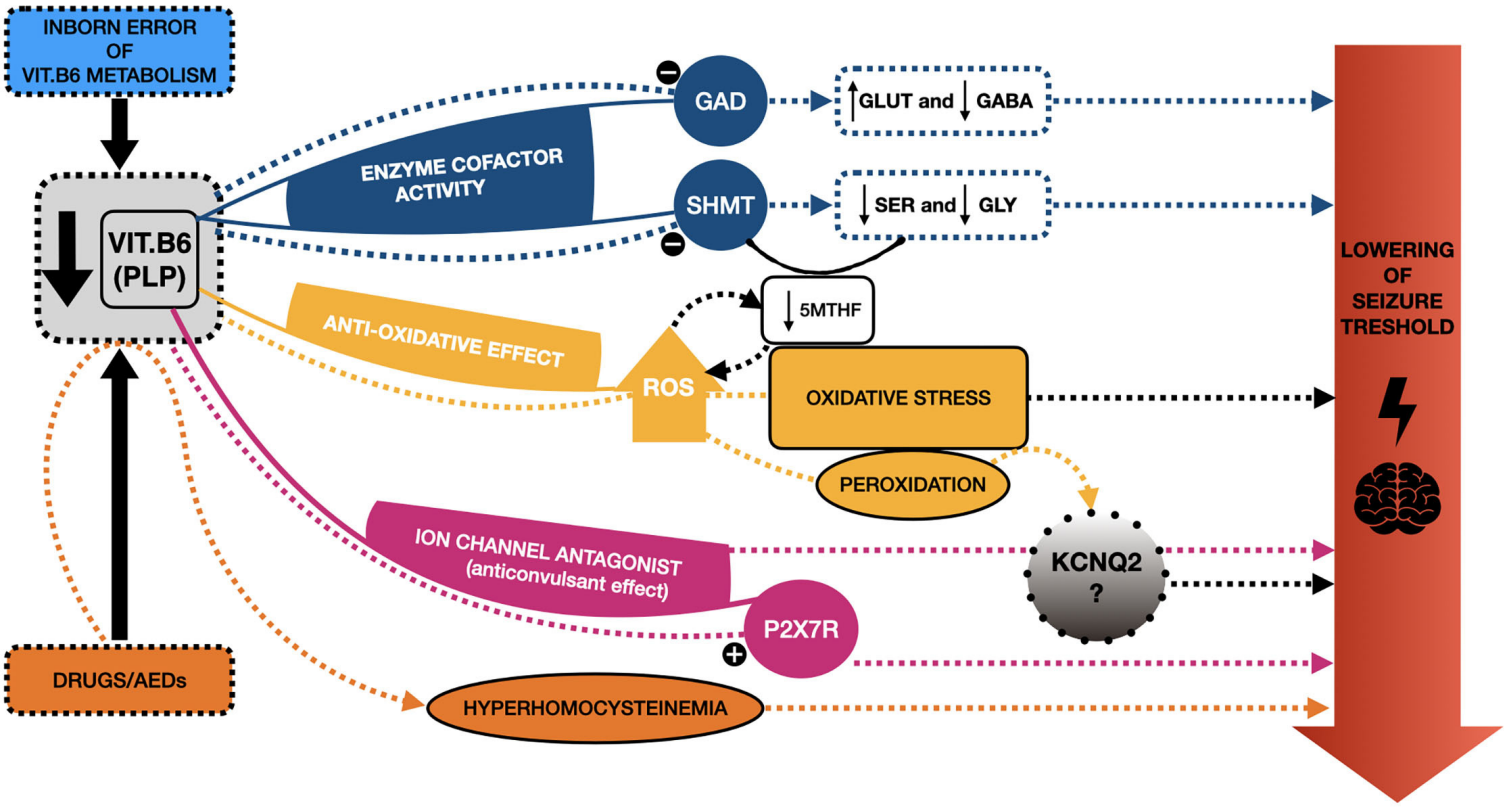
Schematic representation of the mechanisms proposed to explain seizure responsiveness to vitamin B6 and the potential link with KCNQ2. Several etiopathogenic mechanisms have been proposed to explain the responsiveness of certain neonatal seizures to vitamin B6 (VitB6), and particularly to its active form, pyridoxal 5′ phosphate (PLP). PLP acts as a cofactor of several enzymes, such as Glutamic acid decarboxylase (GAD), regulating the synthesis of gamma-amino butyric acid (GABA) from Glutamate (GLUT). A reduction of PLP may determine an imbalance between these two (in favor of GLUT), hence decreasing the seizure threshold. PLP is also involved in the synthesis of serine (SER) and glycine (GLY), modulating the serine hydroxymethyltransferase (SHMT) activity; thus, a reduction of PLP may determine lower levels of these two amino-acids, whose reduction has been related to epileptic phenotypes. SHMT is involved in the metabolic pathway, eventually leading to the formation of the main circulating form of folates, the 5-methyltetrahydrofolate (5-mTHF), a reduction of which has been reported in some patients suffering from pyridoxine-dependent epilepsies (PDE). Furthermore, the reduction of 5-mTHF may fall into another proposed mechanism underlying epilepsy, namely oxidative stress, with excessive production of reactive oxygen species (ROS). This may be a consequence of low levels of VitB6 (anti-oxidative agent) and, at the same time, a cause of folates depletion. Additionally, ROS contribute to the peroxidation of several molecules, also affecting ion channels, hence potentially KCNQ2. PLP also acts as an antagonist of specific ion channels, as P2X7R, with a possible anticonvulsant effect, which could not occur when PLP levels are low. A similar antagonist action on KCNQ2, with possible analogous effects, has been proposed. Finally, some drugs, especially antiepileptic ones (AEDs), may be responsible for the depletion of VitB6 and, indirectly, hyperhomocysteinemia, which is known to lower the seizure threshold. Overall, these mechanisms may explain the responsiveness of some neonatal seizures to VitB6 treatment and help shed light on the potential link with KCNQ2-related epilepsies.

Currently, the etiopathogenic mechanism underlying the PN responsiveness of certain neonatal seizures, and particularly of those KCNQ2-related, remains unclear. However, some hypotheses have been proposed [(18); Figure 2].

First of all, VitB6, particularly as PLP, is notably implicated as a cofactor of hundreds of enzymatic reactions, having a role in several functions, such as in the metabolism of amino-acids and the synthesis of neurotransmitters (42). In this regard, PLP is notably a cofactor of glutamic acid decarboxylase (GAD), an enzyme involved in the synthesis of gamma-amino butyric acid (GABA) from glutamate (GLUT). These two are, respectively, the key inhibitory and excitatory neurotransmitters of the CNS, and an imbalance between them has long been considered among the molecular mechanisms underlying epilepsy (43). The reduction of GABA deriving from low PLP levels has been proposed as a potential mechanism in the genesis of PDE, but it is widespread among the authors the opinion that this cannot be the only one (43–45). Ramos et al. (45) have recently highlighted that conflicting data have been reported on GLUT and GABA levels in the CSF of patients suffering from VitB6 deficiency, supporting this hypothesis (45). In this regard, they carried out a study on a model system of VitB6-deficient Neuro-2a cells, revealing a significant reduction in the *de novo* synthesis of serine and, consequently, glycine, whose reduction has been related to epileptic phenotypes, suggesting a potential role of these two (45, 46).

Additionally, it is noteworthy to mention that glycine synthesis from serine depends on the enzyme serine hydroxymethyltransferase (SHMT), which requires PLP as a cofactor. SHMT is involved in the metabolic pathway which encompasses the enzyme methylenetetrahydrofolate reductase (MTHFR) as well, eventually leading to 5-methyltetrahydrofolate (5-mTHF) formation, the main circulating form of folate (45). This is particularly interesting since low levels of 5-mTHF in CSF have already been reported in our second patient and some PDE patients (18, 47).

In particular, the patient described by Reid et al. (18) and ours reported a positive response to VitB6 treatment, both in the presence of low folates and of different isolated lab findings suggestive for VitB6 disorders (respectively, a high plasma-to-CSF PLP ratio in the former, and an elevation of pipecolic acid both in serum and urine in the latter), though in the absence of a genetic confirmation of inborn errors of VitB6 metabolism. Although these findings cannot be fully explained, and no clear evidence on the topic is yet available, all this suggests not only a potential role of folates in PREs and PDEs, but also that there is much more to find out.

Besides, when it comes to folates, it is not possible to state whether their involvement in PDEs/PREs, and, in general, in epilepsy may be a causative factor, a consequence, or both. Their involvement in these conditions may be mutually related to another proposed mechanism underlying epilepsy, namely the excessive production of reactive oxygen species (ROS) (17, 18). Several studies have demonstrated an excessive ROS production in epilepsy, so they outgrow the capability of endogenous antioxidants to contrast their effect (43). Besides, the excess of ROS may determine a depletion of folates, which are known to exhibit antioxidant functions acting as ROS scavengers (18, 48).

Overall, high levels of ROS may have detrimental effects, including the peroxidation of structures and molecules (i.e., enzymes and components of cell membranes), potentially affecting ion channels indirectly as well (43). Moreover, given the demonstrated anti-oxidative effects of VitB6 (49) and the potential effect of ROS on ion channels, one could speculate that there may be a close, albeit yet unknown, relation between VitB6 and channelopathies, such as KCNQ2-related ones.

Anyhow, when ROS production is excessive, the result is a strong oxidative stress and an imbalance favoring the excitotoxicity (43).

Another possible mechanism may involve the recently demonstrated antagonist action of PLP toward P2X-receptors (and particularly the subtype P2X7R) (50). These receptors are a class of ligand-gated ion channels, activated by ATP, contributing to neuro- and glio-transmission and lately associated with epileptic conditions, such as status epilepticus (51). Since P2X7R antagonists have recently been reported as having anticonvulsant effects, this may apply as well to PLP, explaining at least partly its role in controlling seizures (52).

Given the above, Reid et al. (18) proposed that PLP may as well have a direct antagonist effect on ion channels, including the one encoded by KCNQ2. However, further evidence is needed to confirm this hypothesis.

Other potential mechanisms explaining PDE and PRE may relate to secondary defects of VitB6, for instance drug-induced ones. Several drugs, such as carbamazepine, phenytoin, and PB, may reduce VitB6 levels and indirectly lead to hyperhomocysteinemia (which is known to lower seizure threshold) (53, 54). It is likely that the same effect may apply to other AEDs and that it may partly explain the VitB6 responsiveness of certain patients, even when temporary. Unfortunately, it is not possible to confirm or deny this hypothesis for our patients.

Our study presents some limitations, such as its retrospective nature, with consequent possible information biases and lack of specific data (such as details about treatments and diagnostic assessment taking place in different centers from ours, or the correlation between AEDs modification and concomitant levels of VitB6 and homocysteine), as well as the small sample of patients, making it difficult to generalize our findings.

In the light of the above, further studies focusing on the correlation between types of AEDs used, treatment duration, concomitant plasma levels of VitB6, homocysteine and folates, and possibly considering the eventual disease-causing variants associated (and in particular KCNQ2 ones), may help unravel new evidence on the topic.

Prospective studies are needed to analyze and compare the effects of standardized treatment protocols with VItB6 in neonatal epilepsies in general and in those KCNQ2-related in particular.

All this is particularly important when considering the widespread resort to VitB6 trials in neonatal epilepsies in NICUs, where this treatment is quickly taken into account, though not devoid of risks. In fact, high doses of PN and PLP have been reported as causative of peripheral neuropathy and liver toxicity, respectively (14, 18). In our opinion, this sets some limits on the advisability of resorting to a blanket VitB6 treatment. Therefore, our suggestion would be to initiate it as soon as possible in all those cases in which clinical features and/or genetic testing and lab findings suggest, or, even better, clearly demonstrate a VitB6 disorder (preferably after excluding liver diseases and nerve conduction defects). Otherwise, we would instead reserve VitB6 treatment for peculiar situations, such as drug-resistant NEEs, including those with an already proven pathogenic KCNQ2 variant.

## Conclusions

Despite the limits of our study, our data contribute to adding new evidence on the potential beneficial effect of VitB6 treatment in KCNQ2-neonatal epilepsies, in apparent lack of inborn errors of VitB6 metabolism. Further studies should be conducted to elucidate the mechanisms underlying the variability of VitB6 effects in these patients, to help discriminate whether to include or not KCNQ2 (besides ALDH7A1 and PNPO) in the genetic testing of neonatal-onset seizures responsive to PN/PLP, and, finally, to define appropriate clinical guidelines and treatment protocols.

## Data Availability Statement

The datasets presented in this article are not readily available due to ethical and privacy restrictions. Requests to access the datasets should be directed to the corresponding author.

## Ethics Statement

Ethical review and approval was not required for the study on human participants in accordance with the local legislation and institutional requirements. Written informed consent to participate in this study was provided by the participants' legal guardian/next of kin. Written informed consent was obtained from the minor(s)' legal guardian/next of kin for the publication of any potentially identifiable images or data included in this article. Written informed consent was obtained from the participant for the publication of this case report.

## Author Contributions

AN and GD conceived planned and supervised the study. GA and AB wrote the first draft of the manuscript. GA, AB, and FC prepared the tables. GA and FC designed the figures. GS, GV, MS, and VS helped supervise the project. All authors contributed to manuscript revision, read, and approved the submitted version.

## Conflict of Interest

The authors declare that the research was conducted in the absence of any commercial or financial relationships that could be construed as a potential conflict of interest.

## Publisher's Note

All claims expressed in this article are solely those of the authors and do not necessarily represent those of their affiliated organizations, or those of the publisher, the editors and the reviewers. Any product that may be evaluated in this article, or claim that may be made by its manufacturer, is not guaranteed or endorsed by the publisher.
