# Supplementary material for: KCNQ2-Related Neonatal Epilepsy Treated With Vitamin B6: A Report of Two Cases and Literature Review
Source: Front Neurol. 2022 Mar 25;13:826225. doi: 10.3389/fneur.2022.826225 (PMC8992372; doi:10.3389/fneur.2022.826225)
Supplement: Supplementary file 1 [file Table_1.docx]

**Table 1:** Patients described in the literature who carry a KCNQ2 mutation and have been trialed with Vitamin B6.

| **References** | **Patient** | ***Phenotype*** | ***Mutation*** | ***Sex*** | ***Perinatal and early history*** | ***Seizure onset*** | ***Seizure features*** | **AEDs administered**  **(response)** | **Type of Vitamin B6/Response** | **EEG** | **Neuroimaging** | **Seizure and Clinical Outcome** | **Additional Information** |
| --- | --- | --- | --- | --- | --- | --- | --- | --- | --- | --- | --- | --- | --- |
| *Our patients* | **1** | **Pyridoxine responsive epilepsy** | c.1639C>Tp.Arg547Trp  Paternally inherited heterozygous mutation | M | Normal | 2 d | Clonic szs at the limbs, later associated with perioral cyanosis revolving eyes and buccal automatisms (sucking) | MDL (acutely effective)  PB, VPA  (partially effective) | PN i.m.100mg/d, (later switched to oral mainteinance therapy)/ Successful electro-clinical response | 4 d: polyspike wave complexes  4 mo: slight abnormalities of electrical brain activity in the left posterior areas  After PN initiation: disappearance of the EEG abnormalities | MRI (4 d): normal  MRI (4 mo): normal | Sz-free after PN start  5 mo: recurrence of a unique epileptic sz during an intercurrent infectious episode.  No further szs occurred thereafter, either during infectious episodes.  Today (6 ys): still sz-free (only takes daily multivitamin B complex);  global DD; mild autism-like features | Family history positive for epilepsy both in the maternal and paternal line. Father with a KCNQ2 related-epilepsy (treated with VPA andPB).  The same KCNQ2 variant had already been described by Zara et al. (2013) as causative of a BFNE in a female patient with a maternal inheritance.  ALDH7A1 sequencing: negative.  No further genetic testing was performed. |
|  | **2** | **Pyridoxine responsive epilepsy** | c.740C>Tp.Ser247Leu  De novo heterozygous mutation in exon 5 | F | Normal | 2 d | Myoclonic szs associated with sudden loss of muscle tone and rolling eye movements | PB (acutely ineffective)  Several AEDS at onset (ineffective)  VGB,FA (in addition to PLP – effective)  CBZ (maintenance therapy) | PN/ Unsuccesful  PLP (start at 36 d, p.o. 500mg/d in 4-6 doses)/ Immediate sz control up to 18 mo | 2 d: suppression-burst pattern  19 mo: multifocal epileptic discharges  Subsequent interictal EEGs: marked diffuse abnormalities up to a quasi-periodic epileptiform pattern (despite good sz control) | MRI (2 d): normal | Sz-freedom for 18mo (after PLP start at 36 d)  19 mo: occurrence of epileptic tonic spasms both during sleep and wakefulness, later controlled thanks to additional FA to PLP and VGB  Today (10 ys): discrete sz-control (1 episode/1-2 ys); she only takes CBZ as maintenance anti-epileptic therapy; presents axial hypotonia and limbs hpertonia; severe global DD (non-verbal, sever intellectual disability); behavioral and sleep disturbances, for which she takes Promazine and LZP | Routine metabolic exams: normal.  Extended metabolic workup: elevation of pipecolic acid both in serum and urine  CSF analysis: low levels of folates.  ALDH7A1 and PNPO sequencing: normal. |
| *Millichap, et al., 2016* | **3** | **EOEE** | c.1009G>A p.Ala337Thr | NR | NR | 5 mo | NR | LEV, CBZ, CLB, CLZ, FLB, PB, VPA (NR)  EZO (szs began todecrease by the second week on 13 mg/kg/d) | PN/ Unsuccessful | Before EZO: neonatal burst-suppression pattern eventually evolving in hypsarrhythmia with occasional periods with organization for a few seconds  At 6mo on EZO: Good organization, occasional slowing or spike waves | NR | Sz began to decrease by the second week after EZO was started  Abnormal development (not further specified) | Sz proved refractory to multiple AEDs and PN as well. EZO gave some results, even if urinary retention was experienced as side effect |
| *Sands et al., 2016* | **4-5**  **(twins)** | **BFNE** | c.1057 C>G p.Arg353Gl | F | Born at 34wks | 2 d | Focal asymmetric tonic posturing, associated with apnea and desaturation; mainly upper limbs with shifting laterality | PB i.v. 40 mg/kg, CLN p.o., DZP i.v.(NR)  CBZ p.o. 10 mg/kg/day (effective) | PN i.v.100 mg/ Unsuccesful | Normal background  Initial electrical decrement, evolving to rhythmic theta | MRI: normal | Both sz-free off meds at 16 ys  Normal development (16 ys) | Hystory positive for neonatal szs (mother, twin, sister) |
| *Mulkey et al., 2017* | **6** | **NEE** | c.601C>T p.R201C | NR | Normal | 1 d | Exaggerated and sustained startle reaction to touch (no EEG correlate) | EZO, VGB, CLZ, FA (NR) | PN, PLP/ NR | Burst-suppression pattern in the neonatal period.  After 1mo: multifocal epileptiform discharges with random attenuation | MRI (1wk): mild cerebral atrophy  MRI (3 mo): hypo-myelination, diffuse brain volume loss, sub-ependymal heterotopias | Sz outcome NR  Profound DD (13 mo)  Died at 13 mo for cardio-pulmonary arrest | Early neurologic exam: axial hypotonia, increased peripheral tone, hyper-reflexia. No  Infantile Spasms.  Chronic hypoventilation required |
|  | **7** | **NEE** | c.601C>T p.R201C | NR | Normal | 1 d | Exaggerated and sustained startle reaction to noise/touch, apnea.  Early tonic szs.  Infantile spasms at 4 mo | EZO, VGB, PB, VPA, PHT, FA, TPM, LEV, ZNS, LOC, CBZ, STM, KD, CBD enriched cannabis (NR) | PN, PLP/ NR | Burst-suppression pattern in the neonatal period.  After 1mo: multifocal epileptiform discharges | MRI (1wk): mild brain volume loss | Sz outcome NR  Profound DD (2 ys) | Early neurologic exam: severe diffuse hypotonia, hyper-reflexia, tremor  Ventilator required at birth, apneas stopped at 3 months, recurrent aspiration pneumonias |
|  | **8** | **NEE** | c.601C>T p.R201C | NR | Normal | 2 d | Stiffening events (not captured on EEG) | VGB, PB, TPM, ACTH, KD, LEV, CLZ, FA (NR) | PN, PLP/NR | Burst-suppression pattern in the neonatal period.  After 1mo: multifocal epileptiform discharges | MRI (1wk): normal  MRI (1y): increased T2 signal in the basal ganglia | Sz outcome NR  Profound DD (3 ys) | Early neurologic exam: severe hypotonia, hyper-reflexia  Ventilator required at birth for 4 d |
|  | **9** | **NEE** | c.601C>T p.R201C | NR | Born at term: bradycardia prior to delivery via caesarian section | 1 d | Exaggerated startle response, apnea | EZO, VGB, CBZ, CLZ, PB, FA, PHT (NR) | PLP/NR | Multifocal burst-suppression pattern in the neonatal period.  After 1mo: epileptiform discharges | MRI (2 and 4 mo): severe hypo-myelination | Sz outcome NR  Profound DD (4 mo)  Deceased | Early neurologic exam: mixed tone, hyper-reflexia  Respiratory failure, rare short periods off the ventilator, caffeine tried with mild effect |
|  | **10** | **NEE** | c.602G>A p.R201H | NR | Normal | 1 d | Myoclonic spontaneous movements, exaggerated startle to noise/ touch  Infantile Spasms at 2 months | VGB, CBZ, ZNS, PB, FA (NR) | PLP/NR | 3mo: multifocal epileptiform discharges, hypsarrhythmia | MRI (3mo): Hypo-myelination, diffuse brain volume loss | Sz outcome NR  Profound DD (4 ys and 6 mo) | Early neurologic exam: axial hypotonia, increased peripheral tone, hyper-reflexia  Recurrent aspiration pneumonias |
| *Sharawat et al., 2018* | **11** | **NEE** | c.835G >A p.Gly279Ser  Likely pathogenic heterozygous  Missense variant in exon 6 | M | Normal | 7 d | 7 d: repeated szs with up-rolling of eyeballs, generalized stiffening of body and cry (30-60 s each – 15-20 episodes/d) | PB (sz-free for 3 months in combination with PN).  CBZ 20mg/kg/d (sz-free within a week) | PN  started at 15mg/kg/d, later increased to 50mg/kg/d / Partial response | Initial EEG: Burst-attenutation pattern  Repeat EEG: normal | MRI (28 d): normal | Sz-free at 3mo (after PN start)  5 mo: re-occurrence of 1-2 sz/mo  1y: 8-10 sz/d with versive deviation of eyes to either side, stiffening of all limbs (1 mn long)  1y+1wk: sz-free (after CBZ start)  Sz-free at last follow-up and mild global DD (17 mo) | Partial and temporary response to PN. Sz-freedom achieved with CBZ and PN together. |
| *Spagnoli et al., 2017* | **12** | **NEE** | c.913_915del p.Phe305del  De novo | M | Born full-term Apgar Score 3/10 Urgent caesarean section due to maternal pre-eclampsia and cardio-tocographic abnormalities | 10 hs | 10 hs: versive tonic spasms, ± flushing and desaturation ± focal clonic components | PB, PHT, LEV, MDL (ineffective)  CBZ (sz-free) | PN, PLP/ Unsuccessful | Initial EEG: burst-suppression pattern with synchronous and asynchronous bursts, at times accompanied by tonic spasms  After plural AEDS (9mo): multifocal discharges on an abnormal background | MRI (1 d): normal, except for a thin corpus callosum in its anterior third | Sz-free at 9 mo (after CBZ start)  Severe DD; Convergent Squint; Dysphagia; Severe Spastic-dystonic Tetraplegia (9 mo) | The patient was trialed with FA as well, unsuccesfully |
| *Vilan, et al. 2017* | **13** | **BFNE** | c.1076C>A p.Thr359Lys  Maternally inherited | F | Normal | 1 d | Tonic with cyanosis | PB, CZP, PHT, VPA (NR)  Lidocaine (partially effective) | PN/ Unsuccessful | Ictal aEEG: characteristic  (sudden rise of the lower and upper margins of the aEEG of short duration immediately followed by a marked depression of the aEEG amplitude)  Interictal: continuous normal voltage | MRI: normal | Sz-free at 1.5 mo  Recurrence of frequent Sz after 4 years;  Special education, mild ID, ADHD (13 ys) | Pyridoxine was administered to 8 infants without any beneficial effect. All infants needed ≥ 2 AEDs to control their szs |
|  | **14** | **NR** | c.1955dupC p.Pro652fs  De novo | M | Normal | 2 d | Tonic with cyanosis | PB, MDL, CZP (NR)  Lidocaine (partially effective) | PN/ Unsuccessful | Ictal aEEG: characteristic (“)  Interictal: continuous normal voltage | MRI: normal | Sz-free at 21 d  Recurrence of 2 Sz at 4 ys.  Mild delay in MD (5 ys) |  |
|  | **15** | **NR** | c.1065C>G p.Asp355Glu  De novo | M | Normal | 2 d | Tonic with cyanosis | PB, MDL (NR)  Lidocaine (acutely effective)  PHT (effective) | PN/ Unsuccessful | Ictal aEEG: characteristic (“)  Interictal: continuous normal voltage | MRI: normal | Sz-free at 12 d  No Sz recurrence  Normal outcome (2 ys) |  |
|  | **16** | **NR** | c.2296delC p.Leu766fs  De novo | M | Normal | 3 d | Tonic with cyanosis | PB, MDL (NR)  Lidocaine (partially effective) | PN/ Unsuccessful | Ictal aEEG: not characteristic  Interictal: discontinuous normal voltage | MRI: normal | Sz-free at 14 d  Further outcome unknown |  |
|  | **17** | **NR** | c.1527delA p.Glu509fs  De novo | M | Normal | 24 d | Tonic with cyanosis | PB (not effective) | PN/ Unsuccessful | Ictal aEEG: characteristic (“)  Interictal: continuous normal voltage | MRI: normal | Sz-free at 14 d  Further outcome unknown |  |
|  | **18** | **NEE** | c.830C>T  p.Thr277lle  De novo | M | Normal | 2 d | Tonic with cyanosis followed by focal clonic activity | PB, MDL, CZP, TPM (NR)  LEV and lidocaine (acutely effective)  VPA (effective) | PN/ Unsuccessful | Ictal aEEG: characteristic (“)  Interictal: discontinuous normal voltage | MRI: normal | Sz-free at 1.5 mo  1 febrile Sz at 1 year  Nonverbal, autistic features, delay in MD (3 ys) |  |
|  | **19** | **NR** | c.1657C>T p.Arg553Trp  De novo | F | Normal | 1 d | Tonic with cyanosis followed by focal clonic activity | PB, LEV, MDL (NR)  CBZ (effective) | PN/ Unsuccessful | Ictal aEEG: characteristic  Interictal: continuous normal voltage | MRI: normal | Sz-free at 15 d  No recurrence  Normal outcome (16 mo) |  |
|  | **20** | **NEE** | c.901G>A p.Gly301Ser  De novo | F | Normal | 1 d | Tonic with cyanosis | PB, MDL, LEV (NR)  Lidocaine (acutely effective)  CBZ (effective) | PN/ Unsuccessful | Ictal aEEG: characteristic  Interictal: continuous normal voltage | MRI: increased signal intensity in GP | Sz-free at 15 d  No recurrence  ID |  |
| *Pisano et al., 2016* | **21-32** | **NEE** | 12 patients were trialed with adequate dose of pyridoxine, 7 received pyridoxal-phosphate. At the onset, all patients showed axial hypotonia. During follow-up, cognitive impairment | | | | | | | | | | |
| *Klotz et al., 2017* | **33** | **NEE** | c.1023G>C p.Gln341His  De novo | F | Born at term but small for gestational age (SGA) | 7-8 d | Tonic  with cyanosis. | PB,  LEV (partially effective, in combination with PN) | PN trial: 30mg/kg over 3 d/ No immediate effect on sz frequency or EEG  Consequent PLP trial: 30mg/kg/ Improvement of szfrequency shortly after its start (in combination with PB and LEV) | Initial EEG: discontinuous pattern during sleep and wakefulness with multifocal sharp wave complexes predominantly over the right hemisphere  Later: continuous pattern with more impressive epileptiform activity  Last EEG (date unknown): constant rhythmic background slowing, no physiologic sleep characteristic and multifocal epileptic discharges | MRI (4wks): normal | Sz reduction (once-twice every few wks)  Neuro-cognitive sequelae: muscle hypertonia, adynamia; abnormal MD and DD  Better eye contact, turns from prone to supine and reverse, but she is not able to reach for objects with her hands and presents no appropriate vocalization (11 mo) | No metabolic conditions demonstrated; AASA in urine and CSF, PA in plasma and VB6 vitamers normal in blood and CSF.  No detected mutations in ALDH7A1 or PNPO. Therefore PLP was discontinued and, within 24hs, szs returned, occurring up to 20/day. After reintroduction of PLP szs resolved again within 24 hs. Further switch to PN (again) no change in sz frequency was noted. |
| *Reid et al., 2016* | **34** | **NEE?** | c.629G>A p.Arg210His  De novo | F | Born at 38 wks + 6 by spontaneous labour, after an uneventful pregnancy.  1-3 d of life: two episodes of facial reddening, stiffening and pallor associated with feeding and thus assumed to be reflux | 4 d | Episodes of choking and cyanosis, associated with stiffening after which she became floppy. She presented ‘cycling’ movements of her arms with oxygen saturations dropping to 68%, lasting less than 1 min.  6 wks: szs continued to occur sporadically, beginning with both eyes staring towards the corner of the room, mouth pouting, clonic movements of both limbs and respiratory grunting sounds. | PB, LZP (not effective) PHT, CBZ (partially effective in combination with PN) | PN, PLP/ Successful  2 mo: sz control after 6 d of 0.25 mg of PN (oral drops), at a time when she was also receiving 6.7 mg/kg/day of PHT and 11 mg/kg/day of CBZ | 9 d: some asymmetry with larger amplitude responses on the right and abnormal paroxysmal components.  6 wks: abnormal delta activities and intermittently occurring angular or sharp waves, mainly anteriorly whilst at rest. Conversely, when she cried or had been alerted, the recording was of lower voltage without sharp waves but the content was abnormal.  7 y: A recent EEG has shown a change to a left temporal lobe focus. | MRI (28 d); normal brain structures with appropriate maturation but some increased signal intensity in the subthalamic nuclei around the lateral geniculate nuclei bilaterally | Sz outcome at 7 years: szs presenting only during intercurrent illness  7 y: DD with minimal expressive language (she attends a special school but remains healthy except for szs in the context of intercurrent illness).  Since genetic diagnosis, weaning of PN has commenced and her dose has been halved with no increase in szs. | No mutations detected in ALDH7A1 or PNPO.  High plasma- to-CSF PLP ratio indicative of a vitamin B6 disorder.  A full septic screen including a lumbar puncture was negative.  Series of biochemical investigations demonstrated mild, but likely insignificant, abnormalities of plasma amino-acids.  Urine analysis: mild elevation of multiple amino-acids and organic acid analysis revealed mildly elevated 2-oxoglutarate and pyruvate, interpreted as a possible renal tubule leak or immaturity.  Other analytes found to be high were gamma-glutamyl transferase and alkaline phosphatase, a common finding in children on anti-convulsants. |
| *Allen et al.,*  *2014* | **35** | **BFNE** | c.419_430dupp.Val143_Arg144ins GlnTyrPheVal  Maternally inherited (affected mother) | F | Normal | 4 d | Clonic, mainly, but also tonic szs. Clusters multiple/day or days sz-free. Minor cyanosis (hypoxia) in initial period. Subsequently, mainly tonic with upper limb involvement (lasting 1–4 min), Multiple/day, then intermittent clusters (couple/day), weeks and months sz–free. | LEV (some response, required dose increases).  Other drugs used but ineffective:PB, MDZ, LZP. | PLP (used acutely)/ NR | At onset: bilateral independent high amplitude sharp waves, degree of bisynchrony and periodicity (1 Hz at times) normal background.  3.5 months: normal. | MRI (3 wks): normal | Sz outcome: sporadic breakthrough minor szs  Normal developmental outcome (1 y) |  |
| *Mefford et al., 2012* | **36** | **Pyridoxine-dependent epilepsy?** | 1.5 Mb terminal deletion of the long arm of chromosome 20 | M | Precipitous after a 36 week pregnancy complicated by frequent Braxton-Hicks contractions. | 2 wks | Reddening and tonic stiffening of arms, lasting approximately 1 min.  Initially sporadic but, by 8 wks of age, occurring 4-6/day. | PB 15mg/day (partial response) | PN 100mg/d, later increased to 200mg/d and eventually reduced to 150mg/d/ Good electroclinical response (so that PB was discontinued at 11 months) | Initial EEG: hypsarrhythmia with frequent electro-decremental episodes.  Subsequent EEG, the following day: intravenous administration of 100 mg of pyridoxine, which was followed within 1 min by a 95% reduction in the epileptiform activity.  14 wks: slow with multifocal epileptic activity.  11 mo - Video EEG telemetry: Independent and synchronous bifrontal spike and wave discharges; registration of nocturnal episodes turning out to be confusional arousals from slow wave sleep.  5 y: multifocal epileptiform activity with discontinuous background. | First MRI: Slight delay in myelinization  11 mo: Normal | Sz-free (7 ys)  ID and delay in MD  2 ys: hypotonic, legally blind, developmental level between 9-12 months. Noted to have nightly events of inconsolable crying and screaming developing between 2- and 3 hr after falling asleep and lasting from one to 90 min (nocturnal arousals)  7 ys: resolution of nocturnal arousals, developmental handicaps includingminimal expressive language, and lack of bladder and bowel continence; dysconjugate gaze, rotary nystagmus, axial hypotonia, increased tendon reflexes. | Sequencing of the ALDH7A1 gene did notdetect mutations. |
| *Weckhuysen et al., 2012* | **37** | **NEE** | c.613A>G p.Ile205Val | M | During the last 2 months of pregnancy rhythmical jerking similar to szs.  Subsequent normal perinatal and early development. | 2 d | Generalized tonic with clonic components, lip smacking, back arching, and apnoea. Multiple szs daily. | VGB (initially reduced szsand normalized EEG with 7 wksszfreedom).  MDZ (partially effective).  PB, FA, betamethasone, VPA (all ineffective)  TPM, VGB (effective in combination with PN) | PN/The combination of TPM, VGB, and PN controlled szs. | 7 d: multifocal epilepticactivitymostfrequentlyseenin left temporaland right frontalregions. One szwith nystagmus and intermittent bilateral clonic jerks. Ictal changes showed diffuse attenuationwith multifocal spikes.  9 mo: normal. | CT scan (2 d):subduralhaemorrhage.  Generalized hypodense cerebral parenchyma suggestive of hypoxia(but not confirmed on MRI at 11 d).  MRI (11 d): ↑T2 globus pallidus and thalamus. Normal white matter volume;↑T2 ↓T1 signal in periventricularwhite matter; ↑T2signal parallelingthe posterior limbs of the internal capsules.  MRI (3 years and 6 months): bilateral hippocampal enlargement (L>R) with ↑T2 lefthippocampus | Status epilepticus at 3 mo; Sz -free from 9 mo until 8 ys.  Regression with status epilepticus.  DD (not rolling at 6 mo; walked at 16 mo; 30 single words at 4 ys;  8 ys: follows 2 commands, reads small words. No use of toys. Poor fine motor skills Moderate ID). | Head circumference just above 50^th^percentile (4 years). |
| *Borgatti et al., 2004* | **38** | **BFNE, epileptic encephalopathy, and profound mental retardation?** | c.1620G>A p.K526N  Maternally inherited  (Affected mother and two younger sisters) | F | Born at 40 wks by caesarean section due to podalic presentation | 3 d | Clonicszslasting about 40 seconds. Subsequently right sided clonic and tonic-clonicszswith oro-alimentary automatism. | ACTH (partially effective)  PB, VGB, benzodiazepines, PHT, VPA, CZP, immune-globulin (ineffective) | PN/ NR | Ictal EEG at onset: multifocal epileptiform abnormalities asynchronous over both cerebral hemispheres.  Last EEG (date NR): multifocal and bilateral asynchronous epileptiform abnormalities more evident over the left fronto-temporal areas. | MRI (around 4 mo): thin corpus callosum with moderate white matter reduction and slightly enlarged lateral ventricles | Not achieved complete szscontrol.  Many polymorphic szsa day (mainly tonic, atypical absence, andaudiogenic startles).  Severe spastic tetraparesis and profound ID without any language capability (7 ys and 4 mo) |  |
| *Dedek et al. 2003* | **39** | **BNFE** | p.Ser247Trp | M | Born by caesarean section due to prolonged delivery period and symptoms of foetal distress | 3 d | Left or right head deviation, and upper and lower limb involvement. | ACTH (effective).  PB, PHT ,VGB (ineffective) | PN /Unsuccessful | 8 d: theta–delta activity, synchronous, incomplete sleep spindles and sharp waves.  Ictal EEG: Asynchronous background activitywith suppression-burst patterns and multiple paroxysmalabnormalities with random asynchronous attenuationpatterns.  2.5 ys: background activity is normal, and epileptiform abnormalities are only intermittently evident over the bilateral central regions. | CT (7 d): Normal.  CT (10 d): mild, diffuse white matter hypodensity which was mostly obvious in the supratentorial compartment as well as signs of diffuse oedema.  MRI (18 d): normal  CT (30 d): normal  MRI (41 d): normal | Sz-free ACTH (7 wks – immediate improvement of EEG background activity and a progressive reduction of abnormalities. Szfrequency decreased and szsstopped at age 13 wks).  DD (2 ys and 5 mo):head control and social smiling, but not able to sit without support; muscle hypotonia and dystonic features are present |  |
| *Martin et al. 2014* | **40** | **NEE (Ohtahara Syndrome)** | c.827C>T p.T276I | M | Born at 41 wks by emergency caesarean section due to failure to progress | 1 d | Cyanotic episodes, then more obvious szs(up to 200 per day). Tonic spasms (more than 20 per day) lasting 2-3 minutes. | TPM, DZP, and NZP (fits initially continued, but at 5 months were less severe, ceasing by 17 months).  CZP, VGB, FA (ineffective) | PLP/ Unsuccessful | 1 d: very abnormal with a discontinuous pattern (2-3 seconds of EEG attenuation interrupted by burst of irregular slow/fast and sharp waves, synchronous and asynchronous); few periods of more continuous EEG; focal sharp waves, mainly over the left mid central to parietal cortex.  14 d: predominantly abnormal sleep EEG, characterized by synchronous and asynchronous bursts of irregular slow waves, with spike transients interrupted by 1-2 seconds of EEG attenuation (almost burst-suppression), with multifocal spikes standing out during the periods of EEG attenuation.  The quasi burst suppression pattern persisted until age 3 months, after which the EEG became more continuous in wake and sleep.  20 mo: more stable, dominated by drug-induced fast activity, with no epileptiform discharges.  4 y: though continuous, reoccurrence of multifocal sharp/spike wave discharges, maximal over the right mid temporal cortex, spreading in sleep to the right fronto-central areas. | MRI: generalized mild reduction in white matter bulk with a thin corpus callosum, but otherwise normal. | Sz-free.  Severe DD (4 ys) |  |
| *Numis et al. 2014* | **41** | **NEE** | c.1734 G>Cp.Met578Ile | NR | Born at 34 wks. Lack of visual fixation, decreased spontaneous movements, and axial hypotonia. | 4 d | Tonic head, conjugate eye, and mouth deviation, associated with unilateral tonic abduction of the limbs. Apnoea and desaturationrequiring oxygen administration. | CBZ (effective – sz-free within 2 wks).  PB, LEV, TPM, VGB, CLB, CZP, KD, FA (ineffective). | PN, PLP/ Unsuccessful | Interictal EEG: lack of organization and physiologic features with almost-continuous multifocal epileptiform abnormalities intermixed with random asynchronous attenuations.  Ictal EEG: low-voltage fast activity followed by recruiting spikes or theta rhythms arising mainly from the central regions of either hemisphere, followed by focal spike-wave complexes and prolonged focal or diffuse postictal attenuation. | MRI (20 and 33 d of life (with spectroscopy): progressive diffuse hypomyelination with marked thinning of the corpus callosum; T1 signal prolongation in the lentiform nuclei that normalized on day 33 of life. | Sz-free  Severe psychomotor delay, quadriplegia, axial hypotoniawith appendicular hypertonia, and a tendency to opisthotonic posturing (12 months) |  |
| *Saitsu et al., 2012* | **42** | **NEE (Ohtahara Syndrome)** | c.1010C>G p.A337G | M | NR | 7 d | Tonic szs, vomiting. Complex partial szssince age 5. | High dose PB (sz-free and burst-suppression disappeared).  ZNS (ineffective). | PN/ Unsuccessful | Initial: Burst-suppression | NR | Sz-free after high dose PB  Complex partial szssince 5 ys.  Severe ID, no pyramidal signs, no meaningful words, able to crawl, stand with support |  |
|  | **43** | **NEE (Ohtahara Syndrome)** | c.341C>T p.T114I | F | NR | 0 d | Tremor of the upper extremities then generalised convulsions with cyanosis.  Complex partial szssince age 5. | ZNS (sz-free).  CZP, PHT (ineffective). | PN/ Unsuccessful | Initial: Burst-suppression | NR | Sz free after ZNS  Complex partial szssince 5 ys.  Profound DD,and abnormal MD, spastic quadriplegia, developmental quotient 10, bed-ridden, smiling. |  |
|  | **44** | **NEE (Ohtahara Syndrome)** | c.794C>T p.A265V | M | NR | 1 d | Apnoeic spell, then tonic spasms with right opsoclonus like movement. | ZNS, VPA, CZP, CBZ (ineffective). | PN/ Unsuccessful | Initial: Burst-suppression | NR | Intractable szs  DD, no eye pursuit. Myoclonus at the bilateral upper extremities. |  |
| *Kato et al., 2013* | **45** | **NEE (Ohtahara Syndrome)** | c.650C>Ap.Thr217Asn  De novo | F | NR | 0 d | At onset: Pale face for tens of seconds.  1 d: Eye deviation to left followed by tonic szs(0.5-1/h). | High dose PB (sz-free).  ZNS (ineffective) | PLP/ Unsuccessful | 1 d: Burst-suppression, asymmetric.  Transition to other EEG findings: Diffuse high-amplitude spike-and-slow wave, multifocal spikes. | 2 d: ↑T1 signal on globus pallidus  1 mo: ↑T1 and T2 signal on globus pallidus  6 mo: ↑T2 signal on globus pallidus  2 y: normal | Sz-free after high dose of PB  No head control, no words, social smile, profound DDand abnormal MD, spastic quadriplegia, myoclonus, opisthotonic posture (2 ys). |  |
|  | **46** | **NEE?** | c.794C>T p.Ala265Val  De novo | M | NR | 2 d | At onset: Facial flushing and eye fixation.  3 d: Tonic szs(daily). | DZP, MDL, high-dose PB (partially effective).  VPA (ineffective).  CBZ (successful) | PLP/ Unsuccessful | 5 d: multifocal sharp waves.  Transition to other EEG findings: focal spikes moving loci at each recording. | 7 mo and 2 y: mildly delayed myelination, ↑T2 signal on globus pallidus | Sz-free after CBZ. No sz since 16 mo.  Sitting alone at 6 y, moderate DDand abnormal MD, spastic diplegia with dyskinesia, athetotic/dystonic movement (8 ys). |  |
|  | **47** | **NEE (Ohtahara Syndrome)** | c.794C>T p.Ala265Val  De novo | M | NR | 2 d | At onset: No cry, poor suck, stiffening and arching with eye rolling.  5 d: left-sided szs. | PB, CLB, MDZ, VGB (ineffective) | PLP/ Unsuccessful | 5 d: burst-suppression, brief suppression.  Transition to other EEG findings: rolandic spike waves, normal background activity. | Normal at 0 m. | Intractable szs  DD and abnormal MD.Hypotonia, jerking of left arm, tiffening and arching.  Died at 3mo. |  |
|  | **48** | **NEE (Ohtahara Syndrome)** | c.854C>A p.Pro285His  Maternally inherited | F | NR | 0 d | At onset: poor feeding.  2 d : irritability with hypoxia.  8 d: tonic szs(1-4/day). | VPA (sz-free).  PB (ineffective) | PLP/ Unsuccessful | 12 d: burst-suppression, asymmetric. | 12 d: ↑T1 and T2 signal on globus pallidus  3 mo: ↑T2 signal on globus pallidus | Sz-free after VPA, No szsince 3 months.  DQ 35, rolling-over at 18m, no words, moderate DDand abnormal MD, hypotonic quadriplegia (2 ys). | Mother: idiopathic epilepsy since neonatal age, medicated with VPA |
|  | **49** | **NEE (Ohtahara Syndrome)** | c.881C>T p.Ala294Val  De novo  Domain in protein: Transmembrane domain (S4) | M | NR | 1 w | At onset: convulsion-like movements.  1 mo: asymmetric tonic szs(10/day). | TPM (sz-free).  ZNS, VPA (partially effective)  CZP, PB (ineffective) | PLP/ Unsuccessful | < 1 mo: burst-suppression.  3 mo: evolution to hypsarrythmia. | 3 mo: ↑T2 signal on globus pallidus  9 mo: ↑T2 signal on globus pallidus and moderate frontal atrophy with delayed myelination and thin corpus callosum. | sz-free since 6 months.  Profound DD and abnormal MD; no rolling over, no words, hypotonic quadriplegia, mild motor deterioration (3 ys). |  |
|  | **50** | **NEE (Ohtahara Syndrome)** | c.997C>Tp.Arg333Trp  De novo  C-terminal region | M | NR | 2 d | At onset: tonic szs(status epilepticus).  Transition to partial szs(eyes rolling up). | ZNS (almost sz-free).  VPA, lidocaine (partially effective).  DZP, PB, PHT (partially effective) | PLP/ Unsuccessful | 42 d: burst-suppression, brief suppression.  Transition to other EEG findings: multifocal spikes in F4 and T3-5. | NR | Sz-free. Only one szin 10 ys.  Speaking two-word-sentences, walking alone, severe DD, left upper extremity slightly rigid, tooth-grinding (16 ys). |  |
|  | **51** | **NEE (Ohtahara Syndrome)** | c.1689C>Gp.Asp563Glu  C-terminal region | F | NR | 1 d | At onset: Poor feeding with cyanosis.  3 d: tonic szsfollowed by facial clonicszs(>10/day).  Transition to tonic szsor generalised tonic-clonic convulsions (1/week). | CBZ and CZP (sz-free).  PHT, PB (partially effective)  VPA, NZP (ineffective) | PLP/ Unsuccessful | 4 d: Burst-suppression, asymmetric.  Transition to other EEG findings: frontal continuous fast wave and multifocal spikes. | Normal CT at 0 m (no images obtained). | No szssince 10 y, but relapsed at 24 ys after a year of drug withdrawal.  Walking alone at 22 m, a few words at 4–5 ys, moderate DD with autistic features (24 ys) | Mother: symptomatic localisation-related epilepsy controlled with CBZ since school age. |
| *Milhet al. , 2013* | **52-57** | Six patients described that have been treated with vitamin B_6_ during the first month of life. Responses to each AED not stated. | | | | | | | | | | | |

**Abbreviations**: NR: Not reported - BFNE: Benign familial neonatal epilepsy – EIEE: Early infantile epileptic encephalopathy – EOEE: Early onset epileptic encephalopathy – NEE: Neonatal epileptic encephalopathy – F: Female – M: Male – IV: intravenous - P.O.: per os - S:second(s) -Mn: minute(s) – H(s): hour(s) – D:day/s – Wk(s): week(s) - Mo: month(s) – Y(s): year(s)– CBZ: Carbamazepine – CLB: Clobazam - CF: calcium folinate - CLZ: Clonazepam – EZO: Ezogabine –FA: Folinic acid - FLB: Felbamate –KD: Ketogenic diet – LEV: Levetiracetam – LOC: Lacosamide – LZP: Lorazepam - MDL: midazolam – NZP: Nitrazepam - PB: phenobarbital –– PHT: Phenytoine – PN: Pyridoxine – PLP: Pyridoxal 5’ phosphate - TPM: Topiramate – STM: Sulthiame –VB6: Vitamin B6 - VGB: Vigabatrine - VPA :Valproic Acid – ZNS: Zonisamide -Sz(s): seizure(s) – DD: developmental delay– ID: Intellectual disability – MD: motor development
